# Prevalence of dengue and leptospirosis co-infection in a tertiary care hospital in south India

**Published:** 2018-08

**Authors:** Arun Sachu, Anitha Madhavan, Anu Vasudevan, Jayalakshmi Vasudevapanicker

**Affiliations:** 1 Department of Microbiology, Government TD Medical College, Alappuzha, Kerala, India; 2 Department of Biostatistics, Amrita Institute of Medical Sciences and Research Centre, Kochi, Kerala, India

**Keywords:** Dengue, Leptospirosis, Co-infection

## Abstract

**Background and Objectives:**

Dengue and Leptospirosis were often discussed separately with rash being more common in dengue and jaundice in leptospirosis. But with increasing reports of co-infection, the situation has become worse. The main objective of this study was to look for the presence of both Dengue and Leptospira IgM antibodies in serum samples of patients, presenting with acute febrile illness. Medical records of the co-infected patients were examined to analyse the clinical features and laboratory findings.

**Materials and Methods:**

Serum samples of patients presenting with acute febrile illness were screened for the presence of Dengue IgM antibodies and Leptospira antibodies. Clinical features and laboratory parameters of patients with co-infection were compared with patients having dengue alone. Rainfall data was obtained to look for an association between rainfall and Dengue, leptospirosis and co-infected cases.

**Results:**

Co-infection was seen in 33 (3.4%) samples. There was a statistically significant association between clinical features like rashes, bleeding gums and co-infection. There was a statistically significant association between various laboratory parameters like thrombocytopenia and co-infection. There was positive correlation between rainfall and development of dengue, leptospirosis, and co-infection but it was not statistically significant.

**Conclusion:**

The overall prevalence of co-infection was 3.4%. This study re-emphasizes the fact that dengue and leptospirosis are widely prevalent in south India and clinicians should be aware that co-infection with dengue and leptospirosis is not uncommon.

## INTRODUCTION

Dengue fever is one of the most prevalent arboviral disease in the world and can be caused by any of the four serotypes of the virus (1). The virus is widespread in the tropical and subtropical areas. The 1^st^ epidemic of dengue in India was reported from Kolkata (1963–1964) and since then the epidemiology of the virus has been changing. Leptospirosis is a zoonosis caused by pathogenic Leptospira species (2). The annual incidence of leptospirosis ranges from 0.1–1.0 per 100,000 in temperate regions to 10–100/100,000 in humid regions (WHO, Human leptospirosis, 2003). Dengue is the 10^th^ leading cause of death worldwide with approximately 40% of the world population living in areas at risk for developing dengue. The incidence of both diseases is seen to peak in the monsoon and post monsoon periods. Both the diseases can present as mild febrile illness. While leptospirosis can present in a severe form (Weils disease) with jaundice and organ dysfunction, Dengue can lead to Dengue Haemorrhagic fever (DHF) and Dengue shock syndrome (DSS) (3). Both of these diseases were often discussed separately with rash being more common in dengue and jaundice being predominant in leptospirosis. But with increasing reports of co-infection, the situation has become worse.

The vast overlapping spectrum of signs and symptoms of dengue and leptospirosis makes the clinical diagnosis challenging for treating physicians when the co-infection presents in acute form. It is important to differentiate these two diseases because early antibiotic therapy is crucial in leptospirosis, whereas dengue is treated symptomatically. Increased awareness about the presence of co-infection is necessary, so that a high index of suspicion is maintained and for early diagnosis.

The most common method used for the diagnosis of Dengue in most laboratories is a Dengue IgM ELISA and also an Immunochromatograhic assay for the detection of NS1 antigen. RT-PCR, although an excellent method, is not available in most laboratories (4). The Gold standard method for the diagnosis of leptospirosis is a Microscopic Agglutination test (MAT) but it is time consuming and requires the use of live leptospira species cultures. IgM ELISA is used in most laboratories for the diagnosis of leptospirosis (5).

The main objective of the study was to look for the presence of both Dengue and Leptospira IgM antibodies in serum samples of patients, presenting with acute febrile illness. Medical records of the co-infected patients were examined to find the clinical features and laboratory findings.

## MATERIALS AND METHODS

This was a retrospective hospital based study conducted between January 2016 and December 2016. The target population consisted of patients among all age groups reporting with acute febrile illness (AFI) at Government TD medical college, Alleppey. The study was approved by the ethics committee of the Institute. Serum samples of patients presenting with acute febrile illness were screened for the presence of Dengue IgM antibodies by using the Panbio IgM antibody capture ELISA (MAC ELISA) and Leptospira antibodies by using the SD Elisa Kit in the Microbiology department of Government TD medical college, Alleppey. Exclusion criteria included samples negative for dengue and leptospira IgM and those with equivocal results. Co-infection was defined as patients whose serum samples were positive for both dengue and leptospira IgM. One-hundred age and sex matched patients with isolated dengue fever were analysed. Clinical features and laboratory parameters were determined and compared with patients having co-infection. Rainfall data from January 2016 to December 2016 were obtained from the Indian Meteorological department to look for an association between rainfall and dengue, leptospirosis and co-infected cases over the study period.

### Statistical analysis.

Statistical analysis was done using IBM SPSS 20. (SPSS Inc, Chicago, USA). For all the continuous variables, the results are either given in Mean ± SD, and for categorical variables as percentage. To obtain the association of categorical variables, chi square test was applied. To obtain the relationship between two variables, Pearsons correlation was applied. A P-value < 0.05 was considered as statistically significant.

## RESULTS

A Total of 1280 and 1116 samples were tested for dengue and leptopsira ELISA respectively during the study period. A Total of 974 samples were tested for co-infection. Leptospira IgM was positive in 193 (17.3%) samples and Dengue IgM was positive in 350 (27.3%) samples. As per the definition, co-infection was seen in 33 (3.4%) samples.

### Analysis of co-infection cases.

Among the 33 co-infected cases 18 (54.5%) were males and 15 (45.5%) were females. Mean age of the co-infected cases were 37.4 years. Among the 33 co-infection cases, only one (3%) expired and death was due to multi organ dysfunction.

Association between various clinical features and co-infection were analysed and tabulated in Table 1.

**Table 1.** Association of clinical parameters with dengue and leptospirosis co-infection

**Clinical features**	**Dengue alone (n=100)**	**Co-infection (n=33)**	**p Value**
Arthralgia	19 (19)	7 (21.2)	0.78
Vomiting	26 (26)	14 (42.4)	0.07
Abdominal Pain	16 (16)	10 (30.3)	0.131
Retro-orbital Pain	24 (24)	3 (9.1)	0.061
Bleeding Gums	1 (1)	5 (15.2)	<0.001
Rashes	3 (3)	6 (18.2)	0.002
Hepatitis	5 (5)	0 (0)	0.19
Lethargy	14 (14)	8 (24.2)	0.169

All parameters are presented as numbers (%).

We compared the laboratory parameters among patients with co-infection and patients with dengue alone (Table 2).

**Table 2.** Association of laboratory parameters with dengue and leptospirosis co-infection

**Parameters**	**Dengue alone (n=100)**	**Co-infection (n=33)**	**p Value**
Leucocytosis (>10,000)	4 (4)	6 (18.2)	<0.001
Thrombocytopenia (<100,000)	43 (43)	24 (72.7)	0.003
Elevated ALP (>126)	6 (6)	10 (30.3)	<0.001
Elevated ESR (>25)	5 (5)	9 (27.3)	<0.001
Elevated Creatinine (>2)	1 (1)	5 (15.2)	<0.001
Albuminuria	3 (3)	10 (30.3)	<0.001
Haematuria	2 (2)	7 (21.2)	<0.001
ElevatedALT (>47)	40 (40)	20 (60.6)	0.039
Elevated AST (>53)	53 (53)	15 (45.5)	0.452

ALP-Alkaline Phosphatase, AST-Aspartate transaminase, ALT-Alanine transaminase, ESR-Erythrocyte sedimentation rate. All parameters are presented as numbers (%).

### Association between dengue, leptospirosis, co-infection and rainfall.

**Fig. 1.**
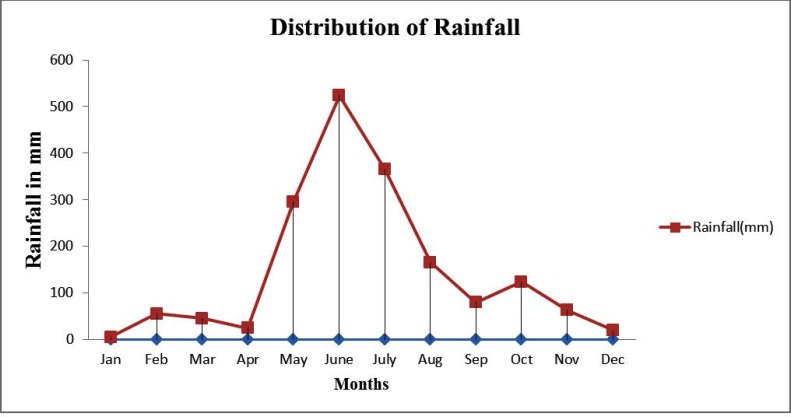
Distribution of rainfall

The distribution of rainfall in Alleppey for the year 2016 is given in Fig. 1.

The rainfall data from January to December 2016 collected from meteorological department show that highest rainfall was recorded from May to October 2016.

**Fig. 2.**
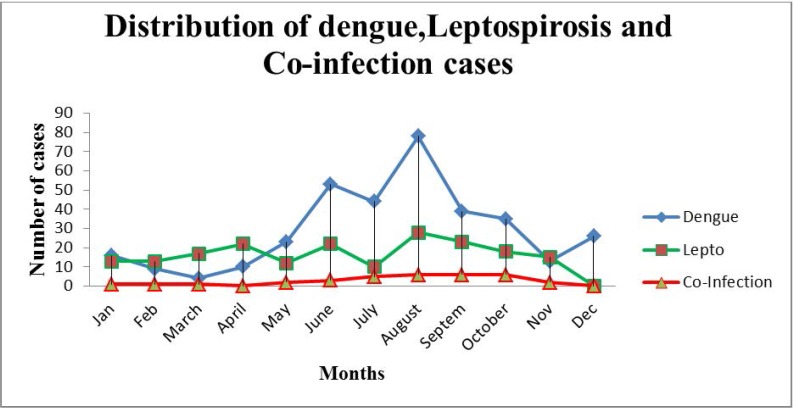
Distribution of dengue, leptospirosis and co-infection cases

Maximum number of dengue and co-infection cases were seen from May to October, while cases of leptospirosis were commonly seen from April to October (Fig. 2). There was positive correlation between rainfall and development of dengue, leptospirosis, and co-infection but it was not statistically significant.

## DISCUSSION

Dengue and leptospirosis are both infectious diseases that have a widespread distribution especially in tropical and subtropical regions (6). They occur more commonly in the rainy season. Dengue is transmitted by Aedes mosquitoes mainly *A. aegypti* and *A. albopictus* (7). Leptospirosis is transmitted mainly by stagnant water containing rodent urine coming into contact with breaks in the skin (8). Detection of leptospira IgM antibody is useful for diagnosis of new infections within 3–5 days of onset of symptoms. Diagnosis of most cases require acute and convalescent testing. Many cases of dengue may be misdiagnosed as a result of inaccurate assessment of disease presentation. Problems of false positivity and false negativity may occur in dengue diagnosis. A positive IgM antibody in late acute or convalescent phase is diagnostic for dengue (9).

Alleppey is the smallest district in Kerala with a very high population density. It is a picturesque town with canals and backwaters which makes it a perfect breeding environment for mosquitoes and rodents. Alleppey experiences a long monsoon season with heavy showers from June to November. According to the National Vector Borne Disease Control Programme (NVBDCP), Tamil Nadu followed by Kerala has accounted for the maximum cases of dengue in the year 2017. Kerala, in fact has seen an alarming rise in the number of dengue cases in 2017 when compared to 2016. Leptospirosis and dengue are common in regions with poor socio-economic conditions. Vast amount of garbage and overflow of wastewater in Alleppey district, attract mosquitoes and rodents, which can in turn spread infections like dengue and leptospirosis (10, 11).

Studies have shown that it is difficult to differentiate leptospirosis from dengue and that leptospirosis is often underdiagnosed in the endemic areas (12). The emergence of co-infection has made the situation even more complicated. This study found serological evidence of co-infection in 3.4% of the samples tested. Studies around the world have reported co-infection ranging from as low as 1.3% to as high as 17.5% (13–16). Kumar et al. reported a mean age of 38 years among the co-infection cases, which is concordant with the findings in our study (14). In this study among the 33 co-infection cases, there was no male predominance. This is discordant with the findings of Suppiah et al. who reported that males are more likely to develop co-infection (17). According to Suppiah et al. there was a statistically significant association between shock and development of co-infection. This was discordant with the findings in our study where there was a statistically significant association between rashes, bleeding gums, leucocytosis, thrombocytopenia, elevated ALT, elevated ALP, elevated ESR, elevated creatinine, albuminuria, haematuria and development of co-infection. Shock was not a significant parameter in our study.

Thrombocytopenia which is a predominant finding in dengue fever, was seen in 72.7% of the co-infection cases. Kumar et al. also reported thrombocytopenia among 64.7% of the co-infection cases (14). Deranged hepatic function was seen among the co-infection cases. Despite serological evidence of co-infection, the mortality in our study was very low (3%). Studies have shown conflicting mortality rates among co-infected cases. Sharma et al. reported a high mortality among co-infected cases (12.69%) (3). Ananthi et al. in her study found that all the patients with co-infection survived (18). Pan Bio IgM ELISA, SD ELISA were used for dengue and leptospirosis, which have shown sensitivity of 85.6% and 70% respectively in other studies (19, 20).

If antibiotics are used to treat leptospirosis, they should be initiated as soon as the diagnosis despite initial serologic results. All the co-infected cases in our study were treated with intravenous crystalline penicillin and fluid resuscitation. A Cochrane review found insufficient evidence to advocate for or against the use of antibiotics in the therapy for leptospirosis (21). Early treatment has been shown to offer the best clinical outcomes. Severe cases can lead to multiorgan failure. Supportive therapy and careful management of renal, hepatic, hematologic and central nervous system complications are important. Dengue fever is typically a self limited disease with a mortality rate of less than 1% when detected early and with access to proper medical care. When treated, severe form of dengue has a mortality rate of 2–5%, but when left untreated, the mortality rate is as high as 20%. Symptomatic and supportive treatment is usually the basic effective management for patients with dengue (22).

In this study we found a positive correlation between rainfall and prevalence of dengue, leptospirosis and co-infection. Similar positive correlation has been found in other studies (23–25). It is important to take into account other factors that affect the transmission of these infections, like relative humidity and temperature, before concluding that rainfall is the reason for the increased transmission of these infections.

## CONCLUSION

The overall prevalence of co-infection of dengue and leptospirosis was 3.4%. There was a statistically significant association between clinical features like rashes, bleeding gums and co-infection. There was a statistically significant association between various laboratory parameters like thrombocytopenia and co-infection. There was positive correlation between rainfall and development of dengue, leptospirosis, and co-infection but it was not statistically significant. This study re-emphasizes the fact that dengue and leptospirosis are widely prevalent in south India and clinicians should be aware that co-infection with dengue and leptospirosis is not uncommon.

## References

[1] Guo RN Lin JY Li LH Ke CW He JF Zhong HJ The prevalence and endemic nature of dengue infections in Guangdong, South China: an epidemiological, serological, and etiological study from 2005–2011. PLoS One 2014; 9(1):e85596. 24465613 10.1371/journal.pone.0085596 PMC3900419

[2] Levett PN. Leptospira. In: Murray PR Baron EJ Jorgensen JH Landry ML Pfaller MA, editors. Manual of Clinical Microbiology. Washington DC: ASM Press; 2007 p. 963–70.

[3] Sharma KK Latha PM Kalawat U. Coinfection of leptospirosis and dengue fever at a tertiary care center in South India. Scho Res J 2012;2:12–16.

[4] Mardekian SK Roberts AL. Diagnostic options and challenges for dengue and Chikungunya viruses. Biomed Res Int 2015;2015:834371. 26509163 10.1155/2015/834371 PMC4609775

[5] Niloofa R Fernando N de Silva NL Karunanayake L Wickramasinghe H Dikmadugoda N Diagnosis of leptospirosis: comparison between microscopic Agglutination Test, IgM-ELISA and IgM Rapid Immunochromatography Test. Dellagostin OA, ed. PLoS One 2015;10(6):e0129236. 26086800 10.1371/journal.pone.0129236 PMC4472754

[6] Libraty DH Myint KSA Murray CK Gibbons RV Mammen MP Endy TP A comparative study of Leptospirosis and dengue in Thai children. PLoS Negl Trop Dis 2007;1(3): e111. 18160980 10.1371/journal.pntd.0000111 PMC2154391

[7] Thenmozhi V Hiriyan JG Tewari SC Philip Samuel P Paramasivan R Rajendran R Natural vertical transmission of dengue virus in Aedes albopictus (Diptera: Culicidae) in Kerala, a southern Indian state. Jpn J Infect Dis 2007;60:245–249. 17881861

[8] Dechner A. A retrospective analysis of the leptospirosis research in Colombia. J Infect Dev Ctries 2014; 8:258–264. 24619254 10.3855/jidc.3123

[9] Chung SJ Krishnan PU Leo YS. Two cases of false-positive dengue non-structural protein 1 (NS1) antigen in patients with hematological malignancies and a eeview of the literature on the use of NS1 for the detection of dengue infection. Am J Trop Med Hyg 2015;92:367–369. 25385858 10.4269/ajtmh.14-0247 PMC4347343

[10] Speelman P. Leptospirosis. Harrison's principle of internal medicine. 14th ed., vol. 1 p. 1036–8.

[11] Peters CJ. Infections caused by arthropod and rodent borne viruses. Harrision's principle of internal medicine. 14th ed., vol. 1 1998 p. 1132–46.

[12] Bruce MG Sanders EJ Leake JA Zaidel O Bragg SL Aye T Leptospirosis among patients presenting with dengue-like illness in Puerto Rico. Acta Trop 2005;96:36–46. 16083836 10.1016/j.actatropica.2005.07.001

[13] Brown MG Vickers IE Salas RA Smikle MF. Leptospirosis in suspected cases of dengue in Jamaica, 2002–2007. Trop Doct 2010;40:92–94. 20305103 10.1258/td.2009.090340

[14] Kumar A Balachandran V Dominic A Dinesh KR Karim S Rao G. Serological evidence of leptospirosis and dengue coinfection in an endemic region in South India. Ann Trop Med Public Health 2012;5:286–290.

[15] Kaur H John M. Mixed infection due to leptospira and dengue. Indian J Gastroenterol 2002;21:206. 12416761

[16] Deodhar D John M. Leptospirosis: experience at a tertiary care hospital in Northern India. Natl Med J India 2011;24:78–80. 21668048

[17] Suppiah J Chan S-Y Ng M-W Khaw Y-S Ching S-M Mat-Nor L-A Clinical predictors of dengue fever co-infected with leptospirosis among patients admitted for dengue fever – a pilot study. J Biomed Sci 2017;24:40. 28659189 10.1186/s12929-017-0344-x PMC5488303

[18] Ananthi B Sumathi G. Concomitant infection of Leptospirosis with dengue at a tertiary care hospital in Chennai. J Evol Med Dent Sci 2014; 3:1823–1827.

[19] Pal S Dauner AL Mitra I Forshey BM Garcia P Morrison AC Evaluation of dengue NS1 antigen rapid tests and ELISA kits using clinical samples. PLoS One 2014;9(11):e113411. 25412170 10.1371/journal.pone.0113411 PMC4239072

[20] Tanganuchitcharnchai A Smythe L Dohnt M Hartskeerl R Vongsouvath M Davong V Evaluation of the standard diagnostics Leptospira IgM ELISA for diagnosis of acute leptospirosis in Lao PDR. Trans R Soc Trop Med Hyg 2012;106:563–566. 22818757 10.1016/j.trstmh.2012.06.002 PMC3464426

[21] Brett-Major DM Coldren R. Antibiotics for leptospirosis. Cochrane Database Syst Rev 2012;(2):CD008264. 22336839 10.1002/14651858.CD008264.pub2 PMC11299142

[22] Singhi S Kissoon N Bansal A. Dengue and dengue hemorrhagic fever: management issues in an intensive care unit. J Pediatr (Rio J) 2007;83:S22–35. 17530136 10.2223/JPED.1622

[23] Sumi A Telan EF Chagan-Yasutan H Piolo MB Hattori T Effect of temperature, relative humidity and rainfall on dengue fever and leptospirosis infections in Manila, the Philippines. Epidemiol Infect 2017;145:78–86. 27608858 10.1017/S095026881600203X PMC9507329

[24] Pappachan M Sheela M Aravindan K. Relation of rainfall pattern and epidemic leptospirosis in the Indian state of Kerala. J Epidemiol Community Health 2004;58:1054. 10.1136/jech.2003.018556 PMC1732650 15547074

[25] Wiwanitkit V. Strong correlation between rainfall and prevalence of dengue in central region of Thailand in 2004. J Rural Trop Public Health 2005;4:41–42.

